# Newborn screening in Karnataka: A scoping review of the landscape

**DOI:** 10.1371/journal.pgph.0007183

**Published:** 2026-09-02

**Authors:** Liz Maria Kuriakose, Diwakar Mohan, Mohua Chakraborty Choudhury

**Affiliations:** 1 DST Center for Policy Research, Indian Institute of Science, Bengaluru, India; 2 Department of International Health, Bloomberg School of Public Health, Johns Hopkins University, Baltimore, Maryland, United States of America; 3 Institute of Public Health, Bengaluru, India; 4 Isaac Center for Public Health, Indian Institute of Science, Bengaluru India; PLOS: Public Library of Science, UNITED STATES OF AMERICA

## Abstract

Newborn screening (NBS) using dried blood spot (DBS) analysis is an important public health intervention to detect serious congenital disorders early, and reduce childhood mortality, and lifelong disabilities. While institutionalized for decades in high-income countries, NBS remains limited in low- and middle-income countries such as India. Despite recommendations in national child healthcare policies, few Indian states have adopted NBS. Even Karnataka, a state performing well in maternal and child health indicators and one of the first states to pilot large-scale NBS studies in the 1980s, is yet to implement a statewide program. This study presents a scoping review of the literature on NBS in Karnataka to understand the NBS efforts so far, identify gaps and provide recommendations for the future. A search of English-language peer-reviewed studies and newspaper articles on newborn/neonatal screening in Karnataka (1980–2024) was conducted using PubMed, Google Scholar, and Google News. Nineteen articles were included, charted using the ISNS framework, and thematically analysed. The results highlighted prevalent themes of research such as disease burden, awareness gaps, inconsistent diagnostic thresholds, consanguinity, lack of data, limited outreach programs, and inadequate NBS facilities. Most studies were hospital-based single-or multi-centre studies limited to the population accessing institutional delivery services. Estimation of incidence and prevalence were the most common research objectives. Diseases commonly considered for screening were inborn errors of metabolism (IEM) and congenital hypothyroidism (CH). A significant burden of CH (ranging from 1:1000) and IEMs (ranging from 1:811) were reported, with certain communities at a higher risk due to prevailing marriage practices. Limited evidence on cost suggests that universal newborn screening is economically feasible, with estimated costs of USD 6.45 per child for a single disorder. All studies support the need for NBS in Karnataka, but further health systems research is needed to inform sustainable implementation.

## 1. Introduction

Newborn screening (NBS) using dried blood spot (DBS) is a set of tests performed shortly after birth to detect disorders in infants before the onset of symptoms. Key to achieving Sustainable Development Goal (SDG) 3.2 [1], NBS within 24–72 hours after birth has proven to be a cost-effective method of early identification of serious disorders [2]. DBS is performed by pricking a newborn’s heel to collect a blood spot on absorbent paper, and is further analyzed in a laboratory. DBS specimens remain stable for a long period and require minimal storage and transportation, enabling a centralized lab to efficiently serve a large region [3]. NBS enables early detection, diagnosis and treatment, which can prevent catastrophic health consequences such as severe physical disabilities and early death. More than sixty years have passed since the beginning of NBS by Dr. Robert Guthrie (1961) in New York [4].

Globally, studies have mentioned that disease priorities need to be reimagined as infectious diseases decline and birth rates fall. This elevates the importance of prioritizing those disease conditions that were previously less common, such as congenital disorders, in healthcare needs and policy [5]. NBS is an important tool to address the increasing burden of congenital disorders through early identification and management. The World Health Organization (WHO) noted that the contribution of birth defects to under-five mortality in the South-east Asian Region (SEARO) has increased from 4% to 11% during the last two decades (2000–2021) even as countries successfully addressed other major causes of death [6].

Since its initiation in 1961, NBS has been successfully implemented for over five decades in most high-income countries such as Norway, Germany, Australia, Canada, Denmark, the UK, Italy, and the USA [4, 7, 8]. However, it has not yet been widely adopted in many low- and middle-income countries, including India [8, 9].

The World Health Assembly Resolution on Birth Defects passed in 2010 endorsed NBS, whereby all member states are encouraged to consider universal NBS programs to screen for birth defects [10]. In response to this, the Regional Director of WHO-SEARO in 2024 released an implementation guide for screening newborns for three select disorders using non-invasive tools [6]. Further, the WHO has urged all member states to adopt, internalize and use the implementation guidance to introduce and conduct a set of screening tests for all newborns before hospital discharge.

NBS as a public health program goes beyond a mere testing activity and includes screening, confirmatory diagnosis, follow-up of positive results, referral to specialist care, and counseling of parents [11]. Selection of diseases to be screened has been primarily guided by the Wilson & Jungner criteria [12]. The disease panel varies across geography and depends on the epidemiology, incidence, and severity of diseases, costs of screening, and availability of treatment [12].

In India, a government-supported NBS program has not been implemented in most states despite many pilot studies conducted since the 1980s. Though 61.9% of births are delivered in public health facilities [13], NBS is now available only in a few high-end private facilities. Looking at the nation as a whole, there are significant barriers that hinder the implementation of a universal NBS program. First, population-based epidemiological studies showing the exact burden and incidence of childhood disorders in different parts of the country are unavailable, which impedes the selection of a disease panel. Second, there is very low awareness about treatable genetic disorders, NBS, and availability of technologies for screening among the medical community [14, 15]. Third, lack of quality infrastructure and trained manpower for NBS and treatment of cases diagnosed further constrains the scope of a universal program [15, 16]. Last, differing national priorities, the absence of a national policy, guidelines, and funding disrupts the scope of a universal NBS in the country [14, 15].

In 2011, the National Neonatology Forum in India put forth a set of recommendations for NBS, which included a group-wise screening strategy comprising of a basic screening panel for three conditions [CH, Congenital Adrenal Hyperplasia (CAH), Glucose-6-phosphate dehydrogenase deficiency (G6PDD)] for all newborns (Group A), a panel for around 12 conditions for high-risk population (Group B), and expanded NBS, including 30–40 disorders classified as inborn errors of metabolism (IEM) in resource-rich settings [17]. Under the Rashtriya Bal Suraksha Karyakram (RBSK), babies born at public health facilities and at home are recommended to be screened for CH, sickle-cell anemia, and beta-thalassemia on an optional basis [18]. However, only a few states/union territories, viz., Chandigarh, Goa, Delhi, and Kerala, have initiated implementation of a statewide public NBS program [19, 20]. Other than Kerala, all the other three states/Union Territories have smaller geographic areas and populations. Therefore, there are not many examples of large-scale statewide implementation of NBS in India.

In this context, Karnataka, one of the more populous states in the country, ranking eighth by population, provides an important setting for examining large-scale efforts. Notably, it was among the first states to undertake a large-scale pilot for NBS of inborn errors of metabolism (IEMs), demonstrating early leadership in the field. However, despite this pioneering effort, progress toward sustained, statewide institutionalization has remained limited, highlighting a gap between early innovation and long-term program integration. One of the earliest attempts at NBS for IEMs was launched as a large-scale pilot study in the state of Karnataka wherein 98,526 neonates were screened in 50 hospitals and maternity homes in Bangalore and Mysore for a period of eight years (1980–1988) [21–24]. Despite being the pioneer state to pilot a large-scale NBS, Karnataka has not implemented any government policies on NBS so far. In recent years, only a few hospital-based NBS programs have been initiated, and some private hospitals and laboratories offer commercial NBS services [20]. Therefore, it becomes crucial to understand why despite an early start, Karnataka lags in implementing NBS as a public health program.

Karnataka has a population of around 67 million, comparable to that of the United Kingdom. The state reports a birth rate of 16.5 per 1,000 population [25], and ranks 22nd out of 35 states/Union Territories (in descending order) in neonatal mortality of 15.8 per 1000 live births [26]. According to the fifth National Family Health Survey (NFHS), 64.8% of births are reported as delivered in public health facilities in Karnataka [26]. In this backdrop, the objective of the review is to understand the status of NBS in Karnataka and the initiatives for NBS that have been taken so far and identify the challenges and recommendations in NBS implementation highlighted by earlier studies. This will be useful for researchers, public health professionals, and policymakers to develop a strategy for NBS implementation in this state and more widely for other states as well.

## 2. Methodology

This scoping study aims to rapidly map the key concepts underpinning the newborn screening research area and the main sources and types of evidence available in Karnataka [27]. This scoping review was based on the methodological framework given by Arksey & O’Malley and Levac et al., and further refined by the updated Joanna Briggs Institute guidance [27–29]. This study followed the PRISMA-ScR guidelines for completing this scoping review [29], given in S1 PRISMA Checklist.

### 2.1. Protocol and registration

The authors developed the protocol for this scoping review a priori. Since the commonly used registry, PROSPERO, does not accept scoping reviews, the protocol was not registered. A full protocol is given in S1 Table, and further details will be available from the authors on request.

### 2.2. Eligibility Criteria

The study selection was guided by the following inclusion and exclusion criteria:

**2.2.1 Inclusion criteria**

- Publications dealing with the screening of newborns in Karnataka and related topics such as factors necessitating NBS in the state.
- Included dried blood spot-based method of NBS
- Published in peer-reviewed journals and newspapers that were either openly accessible or could be accessed through institutional subscriptions of the author.
- Publications published between 1980 and 2024 in English.

**2.2.2 Exclusion criteria**

- Books, book chapters, government documents, and conference proceedings on newborn screening in Karnataka were not found and, hence, not included.
- Publications focusing on screening of newborn children for diseases via non-biochemical tests such as ocular or hearing, pulse oximetry, anthropometry, etc. in Karnataka.
- Studies covering the screening of children over 28 days old.
- Publications focused on pregnancy/prenatal screening.
- Publications that covered locations not in Karnataka.
- Publications that were not written in English.

Government documents were excluded due to limited availability in the public domain and the need for different appraisal approaches, while non-English publications were excluded due to language constraints and concerns regarding comparability and peer-review standards.

### 2.3. Information sources

Different kinds of published documents were considered for this study, including peer-reviewed journal publications on empirical studies (qualitative, quantitative, and mixed-methods); program descriptions; case studies; dissertations/theses; and newspaper articles.

### 2.4. Search

A scoping review was conducted to identify studies on NBS programs in Karnataka, India.

The search was limited to availability in the English language. The searches were conducted through the following databases: PubMed and Google Scholar. The following search term was used:

Pubmed: (“Infant, Newborn”[MeSH] OR newborn* OR neonatal) AND (“Mass Screening”[MeSH] OR screening) AND (“India”[MeSH] AND Karnataka[tiab])

Google Scholar: (“newborn” OR “neonatal”) AND (“screening” OR “mass screening”) AND (“Karnataka” AND “India”) Top 200 records screened by relevance.

The literature search was done between 01 July 2024 and 04 December 2024. A detailed search strategy is given in S2 Table.

Google News: (“Newborn screening” OR “Neonatal screening” OR NBS) AND (Karnataka OR Bengaluru OR Bangalore)

### 2.5. Selection of sources of evidence

**Fig 1:**
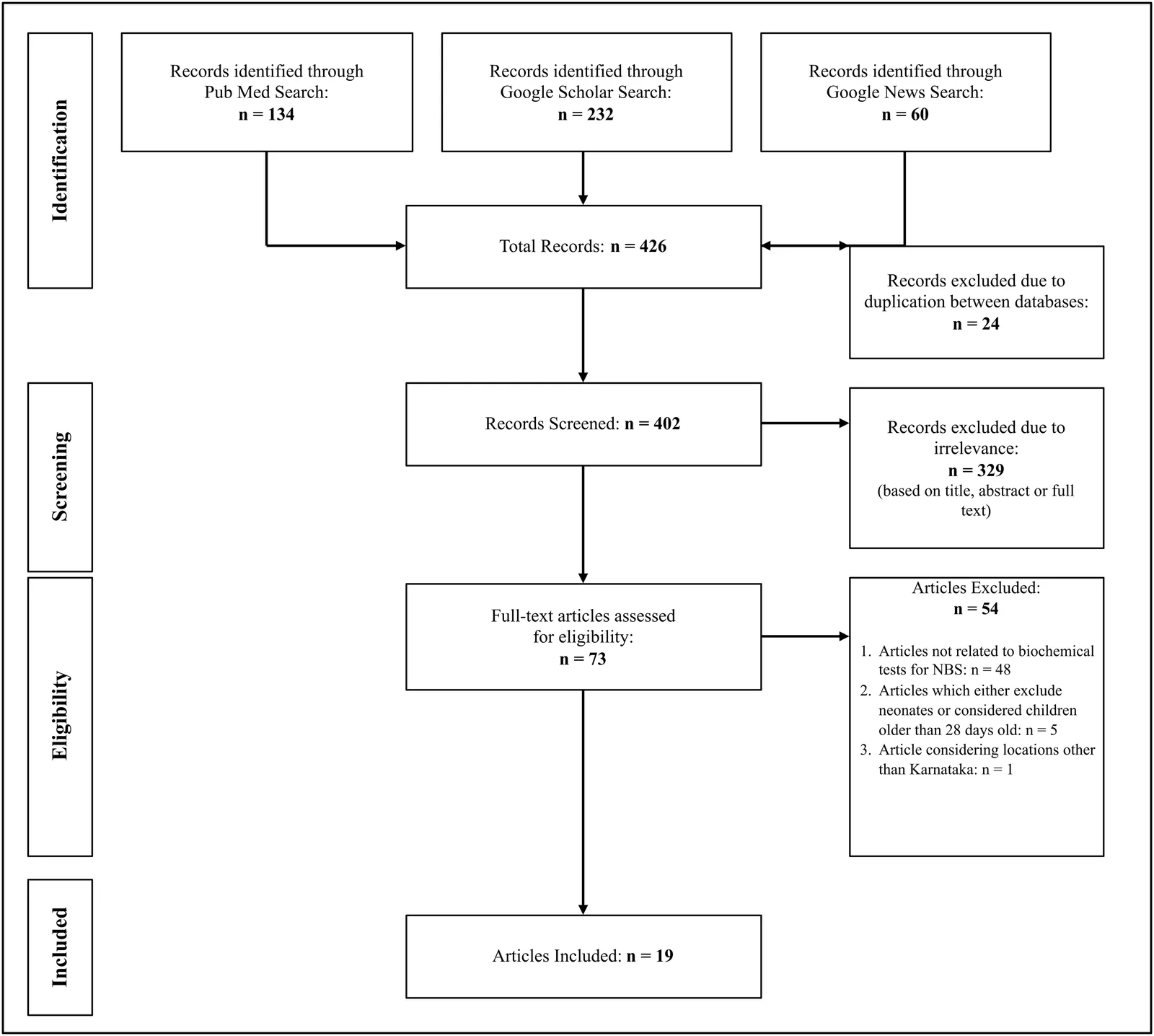
PRISMA Flow diagram on the Selection Process of the Documents for this Review.

The screening process is detailed in the flow diagram, Fig 1, and S1 Table and the search-strategy is given in S2 Table.

The PubMed search extracted 134 records, the Google Scholar search extracted 232 records, and the Google News search extracted 60 records. Thus, a total of 426 records were extracted. 24 records were found to be duplicated in PubMed and Google Scholar, and therefore the redundancies were removed. 402 records were screened, of which 329 records were excluded by screening the title and abstract for not matching the inclusion criteria. This process identified 73 articles for full-text screening, of which another 54 were excluded, mainly for the following reasons: Articles that were not related to dried blood spot-based newborn screening (n = 48), articles that did not consider neonates or considered children older than 28 days (n = 5), or if the study setting was outside Karnataka (n = 21). Finally, **19 articles** were selected which satisfied all the inclusion criteria.

### 2.6. Data charting process

Data were extracted from these studies using the PCC (Population Concept Context) framework to understand the population studied, focus of the research, and context in which it is based. Data charting was conducted as an iterative process to ensure all relevant information was captured. A data extraction template was created in Microsoft Excel and initially tested with five studies. The fields included bibliographic details, methodological characteristics, context, and major themes. The charting form was updated as new concepts emerged during the review process. Two reviewers (MCC and LK) completed data extraction independently, and any disagreements were resolved by consensus.

### 2.7. Data items

The following data were extracted and charted: Bibliographic Information: (Title, Author, Year of Publication, Source Type, Timeline, Site Location); Study Characteristics: (Study Objectives, Study Design and Methods, Sample Size); Newborn Screening Features: (Diseases covered in NBS, Diagnostic Methods of NBS used in study, Sector driving the initiative); Findings from the Study: (Epidemiology Information from the Study, Financial Viability, Socio-cultural Factors); References. S3 Table gives details of all the data items charted in this review.

### 2.8. Synthesis of results

Deductive coding was used to derive major themes, followed by inductive coding to identify the underlying minor themes within some of the major themes. Data from the studies were extracted and charted under the themes in an Excel sheet in S3 Table. The authors LK and MCC did data charting independently and compared, and consensus was reached and reviewed by DM.

## 3. Results

This review considers 19 articles, 13 research papers in peer-reviewed journals, one dissertation, and 5 newspaper reports as listed in Table 1.

**Table 1:** Summary of the reviewed studies that report on NBS screening using biochemical analysis (in chronological order of research).

References	Author name	Disease Condition(s) Targeted	Primary Screening Technique(s) adopted/mentioned	Sample Size	Number of Health care institutions included/ Geographic Location	Timeline of the Research
[21]	Rao et al.	Inborn errors of Amino Acid Metabolism	• Dried blood spot tests from finger prick or toe prick samples • Urine specimens	98,526	Multi-center (50 hospitals and maternity homes)/ Bangalore & Mysore	1980 - 1988
[22]	Bittles et al.	Inborn errors of Amino Acid Metabolism	• Dried blood spot tests from finger prick or toe prick samples • Urine specimens	43,968	Multi-center (50 hospitals and maternity homes)/ Bangalore & Mysore	1980 - 1984
[23]	Bittles et al.	Inborn errors of Amino Acid Metabolism	• Dried blood spot tests from finger prick or toe prick samples • Urine specimens	65,492	Multi-center (50 hospitals and maternity homes)/ Bangalore & Mysore	January 1980 - June 1985
[24]	Devi et al.	Inborn errors of Amino Acid Metabolism	• Dried blood spot tests from finger prick or toe prick samples • Urine specimens	407 infants and children	Multi-center (50 hospitals and maternity homes)/ Bangalore & Mysore	1982-1984
[30]	Babu M et al.	CH	Cord blood samples sent for estimation of Triiodothyronine (T3), Thyroxine (T4) and Thyroid-stimulating hormone (TSH) values by by Chemiluminescence Immuno Assay (CLIA) method	453	Single Centre/ Sri Adichuchanagiri Institute of Medical Sciences, B.G.Nagara	December 2008 - December 2009
[31]	Ramya HS et al.	CH	Whole blood samples	2,376	Single Centre/ Kempegowda Institute of Medical Sciences, Bangalore, Karnataka	June 2011 - May 2013
[32]	Chandrashekhar et al.	G-6PDD	Cord Blood Samples tested using Fluorescent Spot Method	500	Single Centre/ JSS Hospital, Mysuru	July 2012 - January 2013
[33]	Moinuddin, et al.	G-6PDD	Dried Blood Spots analysed by Tandem Mass Spectroscopy	9,136	Single Centre/ Vani Vilas Children’s Hospital, Bangalore	January 2016 - September 2016
[34]	Bhandar, et al.	CH	Intravenous Blood samples	3,150	Single Centre/ Department of Paediatrics, Sangameshwar and Basaveshwar Teaching general hospital attached to Mahadevappa Rampure Medical College, Kalaburagi	December 2014 - September 2016
[35]	Nasheeda, et al.	CH	Cord Blood samples; Newborn canine thyroid-stimulating hormone (cTSH) assay was done by chemiluminescence	1,200	Single Centre/ KS Hegde Medical Academy, Mangalore	18 months
[36]	Kommalur et al	CH, CAH, G-6PDD, GALT & PKU	Dried Blood Spot testing from heel prick samples	41,027	Single Centre/ Vani Vilas Children’s Hospital, Bangalore	January 2016 - December 2018
[37]	Patil P et al.	CH	Heel prick samples analyzed for serum TSH and thyroxine (FT4) levels by CLIA method	3,420	Single Centre/ BIMS, Belagavi	May 2017 - May 2019
[38]	Shetty et al.		To understand the role of maternal awareness and perception in implementation of NBS for inborn errors of metabolism (IEM).	35 mothers	Tertiary care hospital in Kasturba Medical College, Manipal, Karnataka	
[39]	Raveendran et al.	CH, CAH, G-6PDD, GALT, BTD, PKU	Dried blood spot tests from heel prick samples	3,514 in Phase 1; 4,678 in Phase 2	Kasturba Hospital, Manipal, Dr TMA Pai Rotary Hospital, Karkala and TMA Pai Hospital, Udupi in Phase 1; All Government Maternity Hospitals in Udupi (Taluk general Hospital, Kundapura Taluk, Government Hospital, Karkala Taluk, and at the Maternity Hospital, Udupi) in Phase 2.	Phase 1: August 2017 - November 2018 Phase 2: April 2019 - March 2020

The data extracted from the articles and reports used in the study are charted in S3 Table, guided by the PCC framework and categorized into two major groups: (i) study characteristics and (ii) findings from the studies.

### 3.1. Study Characteristics

The characteristics of the studies included for the review are analyzed below:

#### 3.1.1. Timeline.

The timeline of the included articles ranged from 1985 to 2024. As early as the 1980s, three studies, by the same author group, utilized dried blood spot testing for neonatal screening to detect amino acidemias in Karnataka [21–24]. The highest number of articles (44%) were published from 2020 onwards. No studies published between 1990 and 2009 met the review’s inclusion criteria.

#### 3.1.2. Context.

The settings for these studies varied from community (1 study) [33], single hospitals (8 studies) [30–32,34–38] to multi-center collaborations (5 studies) [21–24,39]. Six studies were conducted in the capital city of Bengaluru and the adjacent major city of Mysuru [21–24,31,33]. The remaining studies were based in districts situated outside the capital region, reflecting a broader geographical distribution across Karnataka. Specifically, one study each was reported from tertiary hospitals or medical colleges located in Kalaburagi, Mandya, Belagavi, Manipal, Udupi [30,34,37–39].

#### 3.1.3. Diseases included in the study.

While 7 articles focused on a single disease: CH (5 studies) [30,31,34,35,37] or G-6PDD (2 studies) [32,33], two studies [36,39] and all newspaper articles [40–44] discussed multiple disease screening for NBS such as CH, G-6PDD, PKU, Hyperekplexia, Congenital thyroid abnormalities, Galactose-1-phosphate uridylyl transferase (GALT), CAH, Biotinidase deficiency (BTD), Sickle Cell Anemia, etc.

#### 3.1.4. Study objectives.

The objectives of the peer-reviewed articles (excluding newspaper reports) are categorized into six groups (Table 2).

**Table 2:** Study objectives of the eligible articles included in the review.

S. No.	Study Objective(s)*	Number of articles [Ref]
1	To estimate the prevalence of the disease condition(s) via NBS	2 [33,37]
2	To estimate the incidence of the disease condition(s) via NBS	7 [23,24,31,32,34,36,39]
3	To ascertain the utility of diagnostic tools and/ or techniques	2 [30,35]
4	To estimate the benefit-to-cost ratio of NBS program	1 [39]
5	To investigate the levels of awareness and perceptions towards NBS	2 [38,39]
6	To investigate into the cause-effect of disease condition(s)	2 [23,24]

*Some studies may have more than one objective.

Nine studies focused on estimating the prevalence and incidence [23,24,31–34,36,37,39], while two focused on ascertaining the utility of diagnostic tools [30,35] and one on ascertaining the feasibility of NBS [21]. One study administered pre- and post-awareness structured questionnaires to parents and community health workers [39]. Another developed a counseling module administered to 4,000 mothers in antenatal and postnatal periods [38]. Raveendran et al. performed a cost-benefit analysis in terms of Disability-Adjusted Life Years (DALYs) to understand the cost-effectiveness of the NBS program [39].

#### 3.1.5. Sample size of the studies.

Screening programs included in the study varied significantly in scale. Sample sizes ranged from 35 mothers in a tertiary care hospital study [38] to 98,526 neonates screened in Bangalore and Mysore [21–24]. Other notable sample sizes include 9,136 newborns screened in nine months in 2016 [33], 3,420 screened from May 2017 to May 2019 [37], and 41,027 screened from January 2016 to December 2018 [36]. A two-phased screening program in Udupi screened 3,514 newborns for CH, CAH, G6PD deficiency, PKU, GALT, and BTD from August 2017 to November 2018 and 4,678 newborns for CH and CAH from April 2019 to March 2020 [39].

#### 3.1.6. Study Designs & Methods.

The articles considered in this review varied in their methodology used and included prospective (2 studies) [30,32] and observational (2) [31,33] to cross-sectional (2) [35,37] and retrospective (4) [21–24] studies. Of the 14 peer-reviewed studies, only one had a qualitative methodology [38] (the remaining 12, used quantitative methods).

#### 3.1.7. Diagnostic Methods used for NBS.

Fourteen studies (13 peer-reviewed journal articles and one PhD thesis) deployed biochemical tests involving DBS testing via heel pricks and cord blood samples. These are summarized in Table 1. While cord blood is used for screening CH as observed in two studies, the use of cord blood cells increased false positive rates [30,35] and it was found that this method is not suitable for newborns who have not taken feeds [16]. Screening methodologies varied, with earlier studies (1980–1985) using semi-quantitative thin-layer chromatography [21–24], while a few employed more advanced techniques such as Tandem Mass Spectroscopy to test G6PD [33] and Chemiluminescence Immuno Assay (CLIA) to test T3, T4, and TSH levels [30,31].

#### 3.1.8. Summary of the Reviewed Studies.

The first recorded project on NBS in Karnataka was initiated in 1980 for the detection of amino acid metabolism operating in fifty hospitals and maternity homes in Bangalore and Mysore and tested more than 98,526 neonates over eight years. This was one of the first of its kind NBS studies in India. With a series of publications [21–24], this research project was instrumental in looking into consanguineous marriages and their contribution to the risk of genetic disorders among the offspring of such marriages, as compared to the general population.

Between 2010 and 2019, we found six studies were published, which were largely single-hospital-based studies that focused on estimating the prevalence or incidence of specific metabolic conditions such as CH and G-6PDD [31–34] or the utility of diagnostic methods (such as cord blood TSH to diagnose CH) [30–35].

Studies published between 2020 and August 2024 were a mix of single- and multi-center studies focusing on the prevalence/incidence of metabolic conditions [36,37,39], awareness/perceptions of NBS in the community (especially mothers) [38,39], analysis of benefits to cost ratio of a national NBS program [39], expansion of NBS to government hospitals in partnership with private labs [44], public-private partnership (PPP) in the development of cost-effective rare genetic disorders diagnostics, screening, and treatment [43], private sector collaborations in the newborn and prenatal screening portfolio [41].

The idea of NBS in the public health sector is popularly limited to screening for physical deformities and vision and hearing tests [42].

### 3.2. Major themes identified in the review

The major themes emerging from the studies are discussed below.

#### 3.2.1. Epidemiology.

Primary objective of nine research studies considered in this review was to understand disease epidemiology [23,24,31–34,36,37,39]. Rao et al. detected 46 single and 70 general amino acidemias in the study sample of 98,526 neonates for the period 1980–1988. This study showed a cumulative incidence of 1:847 for different amino acidemias such as hyperphenylalaninemia, tyrosinemia, glycinemia, histidinemia, branched-chain aminoacidemia, and general aminoacidemia [21]. Prevalance and incidence rates varied between different studies. While one study found the prevalence of G-6PDD among all newborns born between January and September 2016 in Vani Vilas Children’s Hospital in Bangalore to be 0.4% (37/9136) [33], another study in a private facility in Mysuru noted zero cases of G-6PDD between July 2012 and January 2013 [32]. The incidence of CH in a single-hospital study at Kalaburagi was reported to be 1:1050 [34]. Similarly, the incidences of permanent and transient CH in a hospital-based study in Bangalore were found to be 1.26 per 1000 live births, and 1.68 per 1000 live births respectively in the year 2014 [31]. Another study in the year 2021 observed a higher prevalence of 4:1000 of CH in their government hospital study in Belagavi [37]. One study reported cumulative incidence rates of IEM conditions in Udupi district from two phases (August 2017 - November 2018 and April 2019 - March 2020) for CH, CAH, G-6PDD, BTD, and GALT as 1:811, 1:2009, 1:932, 1:1475 and 1:1340, respectively [39]. The first large sample size-based study in a government hospital (Vani Vilas Children’s Hospital) estimated the incidence of CH, CAH, G-6PDD, GALT and PKU to be 1:2735, 1:4102, 1:414, 1:41027 and 1:20513, respectively, between January 2016 and December 2018 [36].

#### 3.2.2. Financial Viability of NBS.

Very few articles studied or discussed the cost involved in NBS. Raveendran et al established the viability of running a universal NBS programme after calculating the screening costs for a single disorder at USD 6.45, and screening for each additional disorder at USD 1.5 per child based on the available incidence data [39]. They found that the relative benefits (in comparison to the costs involved) of screening were 6.91, 296.07 and 17.35 times for CH, G-6PDD and CAH, respectively. Using a qualitative study design, Shetty et al gathered the views of 35 mothers who expressed the need for the government to bear the expenses completely or subsidize the cost involved in NBS [38].

#### 3.2.3. Sociocultural factors associated with NBS.

Two important socio cultural factors affecting the newborn screening space as reported in the studies were (i) Consanguity in marraiges and (ii) lack of awareness among all stakeholders including healthcare proffessionals and parents of the newborn.

As reported in the early studies of Bittles et al. and Devi et al. (Bittles et al. 1985; Devi et al. 1987), endogamous marriages and consanguinity are common cultural practices in the population. The coefficients of inbreeding for the single and general amino acidemia groups were found to be 0.0336 and 0.0350, respectively, as compared to 0.0298 as the coefficient of inbreeding in the general newborn population, indicating a possible association between consanguinity and the occurrence of these genetic disorders [21].

Secondly, most studies reported the lack of awareness about NBS among key stakeholders, including parents and medical professionals. At the start of the study in the Udupi district [39], the level of awareness on NBS and IEM among the participants (parents and community healthcare workers) was 30%, which rose to 98% following a series of awareness-creation activities. Some studies reported poor follow-up rates, which are assumed to arise from the lack of sensitization to the deleterious effects of the conditions in the case of a positively screened child [30,35,39]. It has been reported that the parents are more likely to be aware of screening programs only when they have a congenitally diseased elder child or have had incidents of repeated abortions or neonatal death. Improving parental knowledge of these conditions and of screening programs is expected to improve their motivation to bring their children for follow-up and confirmatory evaluations [35].

#### 3.2.4. Government initiatives for NBS.

Five English news articles published between 2015 and 2024 included in this review primarily highlighted the government initiatives around NBS in the state [41–44]. No other relevant articles reported from Karnataka before 2015 could be found from the Google News search. In a chronological order, these articles have reported initiation of free NBS services at a general hospital in Bengaluru [40], private partnerships in neonatal screening [41] to develop cost-effective rare genetic disorder diagnostics, screening, and treatment [43], inadequacy in NBS services provision in the public sector vis-a-vis the private sector [42], and a pilot NBS project in Bruhat Bengaluru Mahanagara Palike (BBMP) six referral hospitals [44].

In 2015, the then Minister for Health & Family Welfare of Karnataka announced that NBS will be provided free of cost at government hospitals, with the responsibility of the whole process being entrusted to a private agency on a turnkey basis, and the scheme to be funded by the National Health Mission. Private hospitals were encouraged to provide NBS at minimum costs determined based on discussions with their administrations. However, there is not much data to substantiate any follow-up implementation of this program in the online public domain. Since 2016, the Indira Gandhi Institute of Child Health has been running a screening program for five disorders at the rate of INR 400 for the above poverty line (APL) category and free of cost for the below poverty line (BPL) patients [42]. Some of the NBS initiatives in Karnataka involve a mix of public and private partnerships wherein samples collected in government hospitals are tested in private laboratories [44].

### 3.3. Challenges reported in the Reviewed Studies

#### 3.3.1. Absence of epidemiological data.

The absence of epidemiological data on the true prevalence and incidence of these disease conditions is noted to be a major barrier [39]. The hospital-based studies are not comprehensive enough to provide a clear picture of the true prevalence and incidence of genetic disorders in the state.

#### 3.3.2. Gaps in awareness.

Gaps in awareness about NBS screening among families and healthcare workers were noted as a major challenge by most of the studies [21,37–39]. Moreover, knowledge of the latest technologies among the healthcare providers was a major barrier to the adoption of the NBS program.

#### 3.3.3. Lack of consensus in cut-off thresholds for disease diagnosis.

Ramya et al mention the controversy and lack of agreement on the cut-off values used to detect CH. They have used a cut-off value of 20 mIU/l for TSH based on the American Academy of Pediatrics (AAP) guidelines [31]. Nasheeda et al follow suit with the same cut-off value [35]. However, Patil et al have used a decreased cut-off value of 10mIU/l, resulting in finding a higher prevalence of cases with CH. They also raise the doubt whether the rising prevalence of CH is real or due to the lowering of cut-off values during screening [37]. Anitha et al screened 41,027 neonates for five disorders (CH, CAH, G6PD, galactosemia, PKU) and found an average recall rate of 0.6%, which meant that 24 normal newborns were recalled for testing for one confirmed case [36]. Rates were higher for G6PD and galactosemia. This study mentioned that “The cut-offs for galactosemia and G6PD deficiency needed modification in order to reduce the high false positives and thereby avoiding higher recall”. Thus, standardizing cutoff thresholds is important for statewide implementation of an NBS program, as it directly impacts the costing of the program.

### 3.4. Recommendations from the reviewed literature

- Dr. Rao in 1988 emphasized that “...as environmental causes of childhood morbidity and mortality decline in the region, so the proportion of diseases with an exclusive or partial genetic aetiology will increase”. Hence, newborn screening becomes an essential public health tool to challenge this new paradigm of childhood diseases [21].
- Population-based estimates of prevalence and incidence of diseases should be done to design a public screening program [34,37,39].
- The unique socio-cultural practices of North Karnataka need to be explored for their interaction with genetic factors and role in human health [39].
- Focus needs to be given in parental awareness to improve the follow-up compliance and confirmatory evaluations [35].
- Diagnostic cutoffs for specific conditions such as CH has to be standardized to reduce the incidence of high false positives and thereby reducing higher recalls [31,37].
- A properly designed awareness program directed towards all concerned stakeholders needs to be done for effective implementation of a newborn screening program in India [38].

## 4. Discussion

All the studies reviewed here have highlighted the need for a statewide NBS program. In the state of Karnataka, socio-cultural factors such as the practice of consanguinity and endogamous marriages have further been correlated with increasing the burden of genetic disorders [21,23]

Findings from this review suggest that even though one of the first large-scale pilot programs for NBS in India was initiated in Karnataka [21–24], the state eventually lost momentum, and a state-wide NBS implementation is yet to be seen. There is a gradual renewal of interest in NBS with individual hospital-based studies. Interestingly, these studies are not only located around the capital region of Bengaluru and Mysuru but also spread across the state in Mangalore, Kalaburagi, Belagavi, and Udupi [34,35,37,38]. Apart from the widely considered factors for NBS such as epidemiology, severity of  disease and cost of  screening [45], the following are the major areas of implementation focus found in this review.

### 4.1. Major areas for implementation focus

**a. Lack of Health Systems Research on NBS**

The NBS program consists of multiple steps as described in ISNS Guidelines for Neonatal Screening [46].However, the existing studies in Karnataka lack critical discussion around the different steps of NBS including its barriers and facilitators at various health system levels. Of the 14 research articles reviewed, nine focused either primarily on estimating the prevalence and incidence of the diseases screened. While no studies focused on implementation areas such as resources required for testing, storage, data management, and research use, public acceptability of the program, and accepted standards of practice to improve the quality of NBS. Research in these areas would help policymakers and health departments to design a sustainable NBS program in the state.

Another major constraint is that NBS is not currently under one umbrella of health services. Facilities for diagnosis and treatment are not easily available at the screening site/referral [15]. Choice of the best mode of blood sample collection, well suited to the local context, is another area that needs to be explored and standardized. This is in light of three reviewed studies using cord blood samples [30,32,34] as opposed to the other studies using bloodspot from heel-prick or toe-stab.

None of the recent articles have delved into qualitative explorations of the social determinants of health in their study contexts that influence these disease conditions, such as consanguinity, female education, social value systems, poor financial conditions, the influence of screening on reproductive decisions, etc.

**b. Ethical challenges**

One of the major ethical challenges identified in this review is the lack of standardized informed consent practices in newborn screening research and programs. Only four studies reported obtaining informed consent from parents before obtaining blood samples for testing [32,35,37,39]. Since newborns are a vulnerable group among human subjects and no standard set of guidelines exists for NBS across the country, it would be ethical to take informed consent from parents and to report it in research publications in the future.

There is a lack of understanding of the social acceptability of this new program in their cultural context, which is very important for planning strategies for successful program implementation. Parental consent and psychological stress involved in the detection of the carrier state, false positive, and false negative results need to be explored at the community level.

**b. Insufficient disease epidemiology data**

Significant variation between epidemiological data was reported in studies from the same state. Only CH screening invariably revealed a significant burden of the disease in the state. Some studies found an average incidence of 1 in 1000 neonates [35], other studies have reported a higher incidence (1 in 13) in the endemic region of the country [47]. Studies on G-6PDD revealed contradicting results, with one study finding no positive cases out of 500 patients [32] and another finding 1 case in 1000 screened babies [33]. Although substantial heterogeneity is reported, disease burden is observed across studies. It is difficult to attribute definitive causes to these variations. While differences in screening cut-off values, study settings, laboratory methods, and population characteristics are often considered as potential contributors, the reviewed studies did not consistently report sufficient methodological detail to allow a systematic comparison or causal synthesis. Nevertheless, a state-wide screening program will generate more reliable data on prevalence even at the pilot phase.

**c. Lack of awareness about NBS**

There is a lack of awareness among parents and healthcare providers. This stems from NBS not being embedded within standard antenatal, intra-partum, and postnatal care pathways, resulting in surprisingly limited awareness not only among parents but among healthcare professionals as well.

**d. Lack of a standard cutoff for the threshold value for disease screening**

Another critical challenge, as reported, is the lack of a standard cutoff for threshold value for disease screening [35]. Lowering cutoffs increases sensitivity but raises recall rates and false positives, burdening families and follow-up services; conversely, higher cutoffs reduce recalls but risk missed cases and false negatives. Thus, a lack of standardized, population-specific screening cut-offs are a major challenge for newborn screening because small shifts in threshold values markedly change program performance. This trade-off has been demonstrated across contexts; for example, reducing the threshold in TSH screening increased case detection of milder CH but also altered the case mix detected [48].

Technical and programatic strategies can mitigate this tension. Population-specific calibration of cut-offs using large, representative DBS datasets (including stratification by age at sampling, birth weight, and feeding status) is essential, as shown in multi-centre tandem mass spectrometry (MS/MS) studies and national efforts to establish local reference ranges. Second-tier testing (e.g., LC-MS/MS or reflex biochemical/genetic assays) markedly reduces false positives and improves positive predictive value without sacrificing sensitivity [48].

For policy and program design, these insights imply two priorities: (1) implement phased, data-driven calibration studies to define locally appropriate cut-offs and reporting standards, and (2) integrate algorithms and transparent methods reporting into routine practice. Combined with ongoing implementation research, these steps will balance sensitivity and specificity while minimizing harms from unnecessary recalls and optimizing resource use [49].

From a scalability and standardization standpoint, the diagnostic methods described above present both opportunities and constraints. Second-round testing and population-specific cut-off calibration can significantly improve diagnostic accuracy but would require advanced laboratory facilities, trained personnel, and quality assurance frameworks that are a major constraint in public health systems. Thus, context-specific, data-driven strategies are needed for sustainable and scalable programs.

**e. Importance of NBS in the presence of consanguinity in Karnataka**

Rao et al’s study was instrumental in highlighting the potential concerns with consanguinity and inbreeding and the availability of presymptomatic screening [21–24]. These studies found that consanguinity is a major risk factor perpetuating genetic disorders in South India, particularly in Karnataka, with a largely negative correlation with socio-economic status [17–20]. Karnataka stands second after Tamil Nadu in the rate of consanguineous marriages (15–49 years age group of married women) at 27% [26]. A public NBS program will help in cross-linking genetic conditions to consanguineous marriages and identifying the actual burden of diseases arising from such unions to raise awareness against such practices.

### 4.2. Learnings from global exemplars

An ideal NBS program could learn from Brazil’s comprehensive approach, which has achieved an impressive coverage of over 82.5% of newborns through 30 reference centers spread across all 26 states and the Federal District [50]. They have established a progressive and inclusive approach, backed by groundbreaking 2021 legislation that mandated comprehensive screening including advanced testing methods like MS/MS for metabolic disorders (amino-acidopathies, urea cycle disorders, fatty acid oxidation disorders), lysosomal storage disorders, and molecular testing for Spinal muscular atrophy (SMA) and Severe combined immunodeficiency (SCID). Their success is built on clear quality indicators, standardized processes for specimen collection, and transparent program documentation through the Ministry of Health. Brazil has further strengthened its program through ongoing research studies focusing on patient outcomes for various conditions (like CH, PKU, and maple syrup urine disease), cost-effectiveness analyses, and continuous evaluation of screening equity, while maintaining flexibility to expand screening panels based on regional needs with the Ministry of Health overseeing a network of strategically placed reference centers across the country [50].

Pilot NBS projects in Brazil provide a strong example of how targeted initiatives can catalyze national program expansion. Designed to assess feasibility, cost-effectiveness, and clinical impact, these pilots integrated advanced screening technologies with robust follow-up systems, leading to marked improvements in early diagnosis and disease management.

In Brazil, states achieving high neonatal screening coverage have largely adopted tandem mass spectrometry (MS/MS), allowing sample collection at maternity facilities and delivering faster, more accurate results [50]. For India, this experience underscores the value of piloting MS/MS in high-burden or high-capacity states to build technical expertise and evidence for scale-up, while acknowledging that nationwide adoption remains constrained by high costs, methodological complexity, workforce training needs, and the challenge of defining population-specific cut-off values.

For Karnataka specifically, Brazil’s experience offers an actionable roadmap: establish a state-level reference center with clear quality indicators and standardized specimen collection protocols; pilot MS/MS technology in high-volume tertiary maternity facilities to build technical expertise and generate local cost-effectiveness evidence; and pursue legislative or policy backing to formalize and sustain the program. Just as Brazil leveraged pilot data to justify national expansion, Karnataka can use evidence from targeted district-level pilots to build the case for statewide scale-up, while drawing on Kerala’s established infrastructure and inter-state data-sharing to accelerate learning and avoid duplicating effort.

### 4.3. Present status and way forward for NBS in Karnataka

The reviewed newspaper articles indicate an interest on the part of the Government of Karnataka towards NBS programs from 2015, but the program faces barriers in terms of a lack of epidemiological data and limitations in implementation [42,44]. Free NBS services have been initiated across six BBMP referral hospitals, which may be expanded. The cost of NBS has been estimated at approximately USD 5 per newborn [19]. Importantly, evidence from six decades across multiple countries demonstrates that NBS is not only cost-effective but also cost-saving in the long term [51].

However, findings from pilot studies conducted in Karnataka included in this scoping review highlight several critical gaps that must be addressed before scale-up. First, there is a paucity of health systems-focused research for NBS, limiting understanding of system readiness, existing capacities, and operational facilitators and barriers for NBS implementation. Second, the studies identify some key implementation challenges, including limited awareness among parents and healthcare providers, insufficient epidemiological data, and the need for population-specific re-calibration of screening thresholds to minimize high recall rates and false positives that could increase system costs. Additional concerns relate to referral pathways, follow-up mechanisms, and workforce workload management.

Collectively, the findings of this review underscore the importance of a phased implementation strategy informed by prior evidence, alongside a strong emphasis on implementation research. Continuous learning from field-level experience and iterative recalibration of NBS processes will be essential to ensure effective, sustainable, and equitable program implementation.

## 5. Limitations of the review

The search for relevant studies could not find government documents in the public domain related to NBS to be considered for this review. The choice of keywords and databases may have constrained the findings. Subjectivity may be present in the process of extracting and analyzing data. It is equally difficult to comment on whether NBS continued to receive the traction it needed, or if it was sidelined between 1990 and 2010. The study is reflective of the developments and trends of screening or technological advancements in NBS in Karnataka, however, it does not claim to have captured every NBS-related initiative in the state due to inherent limitations of data collection methods. The review was limited to only English-language articles, due to which we may have missed important information published in local language, especially in the news papers.

## 6. Conclusion

Overall, this scoping review highlights the limited focus on NBS in Karnataka and underscores the need for greater emphasis on implementing NBS services in the state. Lack of epidemiological data, lack of awareness, and an inconsistent cut-off threshold for disease screening are some of the major challenges highlighted in the studies.

The highprevalence of endogamy and consanguinity and its correlation to genetic disorders further emphasizes the need for Karnataka to implement NBS. Most studies in this review were pilots undertaken in siloes with minimal integration into the public health system. Thus, more evidence-based contextual research on access, case detection, and implementation challenges of NBS from health systems perspectives needs to be conducted to develop a sustainable NBS program in the State. To move towards a sustainable and equitable NBS program, coordinated action is required across multiple stakeholder groups. For state and national policymakers, prioritizing NBS within existing maternal and child health platforms under the National Health Mission, with clearly defined financing and phased scale-up strategies, is essential. For health system planners and program managers, investments are needed in laboratory capacity, referral and follow-up pathways, workforce training, and data systems, alongside re-calibration of screening cut-off values based on population-specific epidemiological data. For clinicians and public health professionals, strengthening awareness, standardized clinical protocols, and timely confirmatory testing and treatment are critical to improving case detection and outcomes. Finally, for researchers and academic institutions, there is a pressing need to generate implementation-focused evidence addressing system readiness, access, acceptability, cost implications, and long-term program sustainability.

## Supporting Information

S1 Table Literature Search. (XLSX)

S2 Table Search Strategy. (XLSX)

S3 Table Data Charting Sheet. (XLSX)

S1 PRISMA Checklist. Preferred Reporting Items for Systematic reviews and Meta-Analyses extension for Scoping Reviews (PRISMA-ScR) checklist.
(DOCX)

## Abbreviations

APL: Above the Poverty Line
BBMP: Bruhat Bengaluru Mahanagara Palike
BPL: Below the Poverty Line
BTD: Biotinidase Deficiency
CAH: Congenital Adrenal Hyperplasia
CH: Congenital Hypothyroidism
CHD: Congenital Heart Disease
DALY: Disability Adjusted Life Years
DBS: Dried Blood Spot
DEIC: District Early Intervention Centre
DNA: Deoxyribonucleic Acid
G-6PDD: Glucose-6-phosphate-dehydrogenase deficiency
GALT: Galactosemia
HIC: High Income Country
IEM: Inborn Errors of Metabolism
IGICH: Indira Gandhi Institute of Child Health
IISc: Indian Institute of Science
LMIC: Low and Middle Income Country
MD: Doctor of Medicine
MS/MS: Tandem Mass Spectrometry
NBS: Newborn Screening
NIH: National Institutes of Health
PKU: Phenylketonuria
PPP: Public Private Partnership
PRISMA: Preferred Reporting Items for Systematic Reviews and Meta-Analyses
RBSK: Rashtriya Bal Suraksha Karyakram
RoP: Retinopathy of Prematurity
SDG: Sustainable Development Goal
TSH: Thyroid Stimulating Hormone
UK: United Kingdom
USA: United States of America
UT: Union Territory
WHO: World Health Organization
SEARO: South East Asian Regional Office
